# Neuroanatomical Substrates and Symptoms Associated With Magnetic Resonance Imaging of Patients With Mild Traumatic Brain Injury

**DOI:** 10.1001/jamanetworkopen.2021.0994

**Published:** 2021-03-18

**Authors:** Sophie Richter, Stefan Winzeck, Evgenios N. Kornaropoulos, Tilak Das, Thijs Vande Vyvere, Jan Verheyden, Guy B. Williams, Marta M. Correia, David K. Menon, Virginia F. J. Newcombe

**Affiliations:** 1 University Division of Anaesthesia, Department of Medicine, University of Cambridge, Addenbrooke’s Hospital, Cambridge, United Kingdom; 2 BioMedIA, Department of Computing, Imperial College London, London, United Kingdom; 3 Department of Radiology, Addenbrooke’s Hospital, Cambridge, United Kingdom; 4 Department of Radiology, University Hospital and University of Antwerp, Antwerp, Belgium; 5 Research and Development, icometrix, Leuven, Belgium; 6 Wolfson Brain Imaging Centre, Department of Clinical Neurosciences, University of Cambridge, Cambridge, United Kingdom; 7 MRC (Medical Research Council) Cognition and Brain Sciences Unit, University of Cambridge, Cambridge, United Kingdom

## Abstract

**Importance:**

Persistent symptoms after mild traumatic brain injury (mTBI) represent a major public health problem.

**Objective:**

To identify neuroanatomical substrates of mTBI and the optimal timing for magnetic resonance imaging (MRI).

**Design, Setting, and Participants:**

This prospective multicenter cohort study encompassed all eligible patients from the Collaborative European NeuroTrauma Effectiveness Research in Traumatic Brain Injury (CENTER-TBI) study (December 19, 2014, to December 17, 2017) and a local cohort (November 20, 2012, to December 19, 2013). Patients presented to the hospital within 24 hours of an mTBI (Glasgow Coma Score, 13-15), satisfied local criteria for computed tomographic scanning, and underwent MRI scanning less than 72 hours (MR1) and 2 to 3 weeks (MR2) after injury. In addition, 104 control participants were enrolled across all sites. Data were analyzed from January 1, 2019, to December 31, 2020.

**Exposure:**

Mild TBI.

**Main Outcomes and Measures:**

Volumes and diffusion parameters were extracted via automated bespoke pipelines. Symptoms were measured using the Rivermead Post Concussion Symptoms Questionnaire in the short term and the extended Glasgow Outcome Scale at 3 months.

**Results:**

Among the 81 patients included in the analysis (73 CENTER-TBI and 8 local), the median age was 45 (interquartile range [IQR], 24-59; range, 14-85) years, and 57 (70.4%) were male. Structural sequences were available for all scans; diffusion data, for 73 MR1 and 79 MR2 scans. After adjustment for multiple comparisons between scans, visible lesions did not differ significantly, but cerebral white matter volume decreased (MR2:MR1 ratio, 0.98; 95% CI, 0.96-0.99) and ventricular volume increased (MR2:MR1 ratio, 1.06; 95% CI, 1.02-1.10). White matter volume was within reference limits on MR1 scans (patient to control ratio, 0.99; 95% CI, 0.97-1.01) and reduced on MR2 scans (patient to control ratio, 0.97; 95% CI, 0.95-0.99). Diffusion parameters changed significantly between scans in 13 tracts, following 1 of 3 trajectories. Symptoms measured by Rivermead Post Concussion Symptoms Questionnaire scores worsened in the progressive injury phenotype (median, +5.00; IQR, +2.00 to +5.00]), improved in the minimal change phenotype (median, −4.50; IQR, −9.25 to +1.75), and were variable in the pseudonormalization phenotype (median, 0.00; IQR, −6.25 to +9.00) (*P* = .02). Recovery was favorable for 33 of 65 patients (51%) and was more closely associated with MR1 than MR2 (area under the curve, 0.87 [95% CI, 0.78-0.96] vs 0.75 [95% CI, 0.62-0.87]; *P* = .009).

**Conclusions and Relevance:**

These findings suggest that advanced MRI reveals potential neuroanatomical substrates of mTBI in white matter and is most strongly associated with odds of recovery if performed within 72 hours, although future validation is required.

## Key Points

**Question:**

What neuroanatomical changes are associated with symptoms after mild traumatic brain injury (mTBI), and when is the optimal time for acute imaging?

**Findings:**

In this multicenter cohort study, 81 patients with mTBI underwent advanced magnetic resonance imaging within 72 hours and 2 to 3 weeks after injury. White matter volume and integrity evolved during that window in tandem with symptoms and were most closely associated with clinical recovery if imaging was performed within 72 hours.

**Meaning:**

These findings suggest that white matter injury is associated with symptoms after mTBI and could, if detected early, help select patients at risk of poor outcome for clinical follow-up or interventional trials.

## Teaser

This multicenter cohort study assesses neuroanatomical substrates in patients with mild traumatic brain injury and the optimal timing for magnetic resonance imaging.

## Introduction

Estimated to affect half the world’s population during their lives, traumatic brain injury (TBI) is a major public health problem and a leading cause of disability.^1^ Based on the level of consciousness on presentation, 70% to 90% of TBI is classified as mild.^1^ This term, however, is clearly a misnomer, because 30% to 50%^2,3,4,5^ of those patients experience symptoms that persist beyond 6 months and disrupt relationships and employment.^6^ Although symptoms may be reduced by early intervention,^4,7^ the large numbers of patients with mild TBI (mTBI) prohibit unselected follow-up without overburdening the system. Similarly, because many patients recover fully, trials of early interventions using unselected populations with mTBI are underpowered. We therefore need ways to enrich populations for clinical follow-up and interventional trials. Magnetic resonance imaging (MRI) offers the potential to improve our understanding of the pathophysiology underpinning patient outcomes and to identify patients at risk of unfavorable recovery.

Results of conventional imaging, including radiographic computed tomography (CT) and structural MRI, are often normal in patients with persisting symptoms and do not explain all of the variance in outcome.^8,9,10^ More advanced MRI, including volumetric analysis and diffusion tensor imaging (DTI), have shown promise in detecting anatomical changes associated with outcome after mTBI, such as traumatic axonal injury.^11,12^ Diffusion tensor imaging characterizes the diffusion of water molecules, which is influenced by the microstructural organization of tissues, offering unique pathophysiological insights.^13^ Studies of mTBI tended to perform imaging at a single point more than 1 week after injury.^8,11^ Changes, however, are likely dynamic in the early phase, and hence the timing of imaging sessions may prove important. This supposition is supported by a systematic review of DTI in the acute to subacute phase,^14^ which found conflicting results, with equal numbers of studies reporting increases and decreases in fractional anisotropy, a marker of white matter integrity. The optimal timing of imaging therefore remains uncertain.

There are no validated outcome prediction models for use in the emergency department. Emergency physician clinical judgment for the estimation of long-term outcomes is overly optimistic, expecting complete recovery in more than 90% of patients, when only approximately 50% achieve it.^15^ Although there are several outcome prediction models for TBI, the well-established ones (CRASH [Corticosteroid Randomization After Significant Head Injury Trial]^16^ and IMPACT [International Mission for Prognosis and Analysis of Clinical Trials]^17^) are primarily aimed at moderate to severe TBI. Two reviews^18,19^ concluded that no available models adequately predict recovery after mTBI. These reviews did not include the prediction model of the UPFRONT study,^3^ which still depends on a psychological assessment at 2 weeks. Thus, an urgent need remains for a tool that risk-stratifies patients early after presentation.

This study investigated 3 questions regarding MRI in mTBI. First, what are the neuroanatomical substrates of mTBI? Second, how do these substrates change with evolving or resolving symptoms? Third, what is the optimal timing for estimating outcomes?

## Methods

### Participants

All eligible patients were included in this cohort study from 2 prospective observational cohorts: the Collaborative European NeuroTrauma Effectiveness Research in Traumatic Brain Injury (CENTER-TBI) study (December 19, 2014, to December 17, 2017)^20,21^ and a Cambridge study (November 20, 2012, to December 19, 2013) with a similar protocol.^22,23^ CENTER-TBI was accessed using the Neurobot platform (RRID/SCR_017004, core data, version 2.0; International Neuroinformatics Coordinating Facility; released May 15, 2019). Ethical approval for CENTER-TBI was obtained in accordance with all relevant laws and regulations for each recruiting site. For the Cambridge cohort, ethical approval was obtained from the local research committee. Informed consent from the patient or legal representative/next of kin was obtained for all participants. Reporting of this study follows the Strengthening the Reporting of Observational Studies in Epidemiology (STROBE) guideline.

Patients sustained an mTBI (Glasgow Coma Score on presentation, 13-15), satisfied local criteria for CT head imaging, and underwent an initial MRI within 72 hours (MR1) and a second MRI within 31 days (2-3 weeks) of injury (MR2). Thirty-nine patients underwent a third MRI (MR3) at 3 months. Across 9 sites, 12 MRI scanners each contributed 6 to 25 healthy volunteers of comparable age and sex who underwent imaging with the same protocol (n = 104).

Demographic and clinical data were collected in the emergency department. Follow-up included the Rivermead Post Concussion Symptoms Questionnaire (RPQ)^24^ at the time of imaging (<72 hours and 2-3 weeks) and the extended Glasgow Outcome Scale^25^ at 3 months.

### Image Acquisition and Analysis

Computed tomographic data were obtained using local site protocols, with no attempt at standardization. Magnetic resonance imaging sequences were acquired at 3 T and included volumetric T1-weighted, volumetric fluid-attenuated inversion recovery, T2-weighted, and susceptibility-weighted imaging and DTI. Base values of DTI were 2-mm isotropic voxels, 32 noncollinear directions, and a *b* value of 1000 seconds/mm^2^ or 2-mm isotropic voxels, 63 noncollinear directions, and a *b* value of 1000 seconds/mm^2^ (Cambridge).^23,26^ Computed tomographic and MRI scans were reported centrally by Cambridge or icometrix investigators blinded to patient outcome based on Common Data Elements and using all available sequences.^27^

Sequences were processed on a TBI-specific pipeline. After neck cropping and correcting for scanner field inhomogeneities, brain parcellation was performed using multi-atlas label propagation with expectation-maximization–based refinement, which provides robust segmentation even when anatomy is distorted owing to trauma.^28^ The 138 anatomical regions were collapsed into 15 regions of interest (ROIs).

All DTI data were corrected for noise,^29,30^ Gibbs ringing artifacts,^31^ head motion and eddy current artifacts,^32^ and inhomogeneities in the magnetic field.^33^ Diffusion tensors were fitted via weighted least squares to derive mean diffusivity and fractional anisotropy maps using the FMBIR Software Library. White matter parcellation into 72 tracts was performed using TractSeg.^34^

Raw data and pipeline outputs for controls and patients were visually inspected by an expert (V.F.J.N.), and motion parameters for DTI were calculated. Outlier values (interquartile range [IQR] >1.5 above the third or below the first quartile) were calculated for fractional anisotropy and mean diffusivity for each scanner, and each tract received particular attention on visual inspection. In the absence of excessive head motion or other artifact, outlier data were retained because they likely reflected true variation or pathology. One patient and 4 controls were excluded from the DTI analysis owing to artifacts.

### Statistical Analysis

Data were analyzed from January 1, 2019, to December 31, 2020, in R version 3.6.0 (R Project for Statistical Computing). Results are reported as median (IQR [interquartile range]) or frequency (percentage), with *P* values before adjustment for multiple comparisons. Statistical significance was determined using a false discovery rate threshold of 5%.^35^ Statistical methods are outlined in eTable 1 in the Supplement. Two-sided *P* < .05 indicated statistical significance.

Within-patient changes between MR1 and MR2 were compared as follows. Lesion presence was compared using the McNemar test. Because total intracranial volume is fixed, an increase in one ROI must precipitate a decrease in another. We therefore chose a compositional data analysis using an additive log ratio.^36^ Univariate analysis was used to identify which ROIs drove this change. For each ROI, the within-patient change was summarized in a single value as log(volume on MR2/volume on MR1), and a 2-sided, 1-sample *t* test was applied. An analogous analysis was performed for diffusion parameters.

Patients were compared with controls as follows. Mixed models were fitted for brain regions that changed significantly between scans. The corpus callosum was also included as commonly implicated in mTBI.^37,38,39,40^ Region of interest volume (normalized for total intracranial volume), fractional anisotropy, or mean diffusivity were the y variables; group (patient vs control), age, and sex, the covariates; and scanner, a random intercept.

The association between scan evolution of DTI and symptoms was assessed as follows. Change in fractional anisotropy was measured as log(fractional anisotropy at MR2/fractional anisotropy at MR1) and similarly for mean diffusivity, bringing both on the same scale. Evolution of DTI was divided into 3 phenotypes using *k*-means clustering. Three clusters were chosen based on biological plausibility, which correlated well with the silhouette (2 clusters) and elbow (3 clusters) methods.^41^ Symptom evolution was measured as the difference in RPQ (RPQ at MR2 minus RPQ at MR1) and compared between phenotypes using analysis of variance.

Logistic regression was used to examine the association between imaging findings and a favorable recovery at 3 months, defined as an extended Glasgow Outcome Scale score of 8. We dichotomized outcome for face validity (patients with mTBI should recover fully), logistic efficiency, and comparability with past studies. Covariates included the number of tracts for which fractional anisotropy, mean diffusivity, or both were abnormal (ie, >2 SD above [for mean diffusivity] or below [for fractional anisotropy] the mean of controls undergoing imaging on the same scanner), as well as age and sex. This binary definition was chosen to reflect vasogenic edema (mean diffusivity) and axonal loss (fractional anisotropy).^42^ It was chosen over a ternary categorization (low/normal/high) because it yielded better model performance and allowed inclusion of terms for “tracts with both mean diffusivity and fractional anisotropy abnormal” without resulting in multicollinarity and overfitting. Models were compared using the area under the receiver operating characteristic curve (AUC), accuracy on 10-fold cross-validation, the Akaike information criterion, and positive (PPV) and negative (NPV) predictive values. In a sensitivity analysis, models were fit after excluding patients with Marshall scores of 5 or 6.

Patients with missing DTI or outcome data were excluded from the respective analysis (eFigure 1 in the Supplement) because adjuvant data were insufficient for multiple imputation.^43^ Characteristics of patients included and excluded from each analysis were compared, and sensitivity analyses were performed (eTable 2 in the Supplement).

## Results

The study included 81 patients (73 CENTER-TBI and 8 local) with a median age of 45 (IQR, 24-59; range, 14-85) years, of whom 57 were male (70%) and 24 were female (30%), and 24 (30%) had a complicated mTBI with a positive initial CT finding (Table 1 and eTable 3 in the Supplement). The incidence of radiology reports with findings positive for lesions was similar at MR1 (n = 34) and MR2 (n = 31), but subarachnoid hemorrhage (9 of 14 [64%]) and intraventricular hemorrhage (8 of 10 [80%]) showed a tendency toward resolution (Table 2). None of these patients underwent neurosurgery between MR1 and MR2.

**Table 1.** Patient Characteristics

Characteristic	Data (n = 81)^a^
Age, median (IQR) [range], y	45 (24-59) [14-85]
Sex	
Female	24 (30)
Male	57 (70)
Mechanism of injury	
Acceleration/deceleration	10 (12)
Blow to head	7 (9)
Fall from height	21 (26)
Ground-level fall	19 (23)
Head against object	11 (14)
Multimechanistic	13 (16)
Glasgow Coma Score	
15	64 (79)
14	12 (15)
13	5 (6)
Injury severity score, median (IQR) [range]	8.5 (4.0-16.2) [1.0-41.0]
Missing	1 (1)
Stratum	
Discharge from ED	42 (52)
Admission for standard care	30 (37)
ICU admission	9 (11)
Recovery at 3 mo^b^	
Favorable	35 (43)
Unfavorable	36 (44)
Missing	10 (12)
Marshall score (pre-MR1)	
1	57 (70)
2	18 (22)
3	0 (0)
4	0 (0)
5	1 (1)
6	5 (6)
Time to MR1, median (IQR) [range], h	36 (25-55) [6-72]
Time to MR2, median (IQR) [range], d	17 (15-21) [9-31]
Time to MR3, median (IQR) [range], d	97 (92-100) [81-120]
Missing	42 (52)

Abbreviations: ED, emergency department; ICU, intensive care unit; IQR, interquartile range; MR1, first magnetic resonance scan after injury; MR2, second magnetic resonance scan after injury; MR3, third magnetic resonance scan after injury.

^a^ Unless otherwise indicated, data are expressed as number (percentage) of patients.

^b^ Recovery at 3 months was considered favorable if the score on the extended Glasgow Outcome Scale was 8.

**Table 2.** Comparison of Lesions Visible on MR1 vs MR2^a^

Abnormality	Scan finding, No. (%) of patients						Raw *P* value
	MR1 positive			MR1 negative			
	MR1 positive	MR2		MR1 negative	MR2		
		Lesion persists	Lesion resolved		Remains negative	Shows new lesion	
Any	34 (100)	31 (91)	3 (9)	47 (100)	47 (100)	0	.25
Mass effect							
Mass >25 mL	2 (100)	2 (100)	0	79 (100)	79 (100)	0	NC
Midline shift	1 (100)	1 (100)	0	80 (100)	80 (100)	0	NC
Cisternal compression	1 (100)	1 (100)	0	80 (100)	80 (100)	0	NC
Intra-axial							
Contusion	20 (100)	20 (100)	0	61 (100)	61 (100)	0	NC
Traumatic axonal injury	21 (100)	20 (95)	1 (5)	60 (100)	60 (100)	0	>.99
Extra-axial							
Hemorrhage							
Epidural	3 (100)	3 (100)	0	78 (100)	78 (100)	0	NC
Subdural	8 (100)	7 (88)	1 (12)	73 (100)	71 (97)	2 (3)	>.99
Subarachnoid	14 (100)	5 (36)	9 (64)	67 (100)	66 (99)	1 (1)	.03
Other							
Skull fracture	0	0	0	81 (100)	81 (100)	0	NC
Intraventricular hemorrhage	10 (100)	2 (20)	8 (80)	71 (100)	71 (100)	0	.01

Abbreviations: MR1, first magnetic resonance scan after injury; MR2, second magnetic resonance scan after injury; NC, not calculated.

^a^ Eighty-one patients received a magnetic resonance scan within 72 hours (MR1) and at 2 to 3 weeks after injury (MR2). Lesions visible on the 2 scans were compared using the McNemar test for paired categorical data. Where reports were identical, no *P* value is shown. None of the *P* values was significant, assuming a 5% false discovery rate.

### Volumetric Analysis

The composition of brain volume changed significantly between MR1 and MR2. This change occurred predominantly in 3 ROIs (Table 3): ventricular volume (MR2:MR1 ratio, 1.06; IQR, 1.01-1.15 [95% CI, 1.02-1.10]; *P* < .001) and circumferential cerebrospinal fluid volume (MR2:MR1 ratio, 1.03; IQR, 1.00-1.14 [95% CI, 1.00-1.07]; *P* < .001) increased, whereas cerebral white matter volume decreased (MR2:MR1 ratio, 0.98; IQR, 0.96-1.00 [95% CI, 0.96-0.99]; *P* = .001).

**Table 3.** Volumetric Changes Between MR1 and MR2^a^

ROI	ROI volume, median (IQR), cm^3^				Raw *P* value^b^	FDR^c^
	MR1	MR2	Absolute difference	Ratio of MR2:MR1		
Cerebrospinal fluid						
Convexity	1.26 (1.11 to 1.63)	1.36 (1.13 to 1.82)	+0.05 (−0.01 to +0.18)	1.03 (1.00 to 1.14)	<.001	Significant
Ventricles	24.59 (17.52 to 33.76)	27.00 (18.72 to 42.27)	+1.49 (+0.12 to +4.20)	1.06 (1.01 to 1.15)	<.001	Significant
White matter						
Cerebellar white matter	16.59 (15.14 to 18.76)	16.38 (14.57 to 18.01)	−0.39 (−1.56 to +0.47)	0.98 (0.90 to 1.03)	.05	NS
Cerebral white matter	231.5 (215.06 to 259.48)	229.85 (210.39 to 253.21)	−3.77 (−9.29 to −0.99)	0.98 (0.96 to 1.00)	.001	Significant
Infratentorial gray matter						
Brainstem	29.01 (26.91 to 30.84)	28.83 (26.46 to 30.5)	+0.08 (−0.26 to +0.43)	1.00 (0.99 to 1.01)	.25	NS
Cerebellar gray matter	61.86 (55.94 to 66.85)	61.82 (58.26 to 67.18)	+0.88 (−0.84 to +2.60)	1.02 (0.99 to 1.04)	.02	NS
Supratentorial gray matter lobes						
Frontal	107.72 (97.9 to 116.55)	108.22 (95.89 to 115.51)	+0.05 (−2.44 to +1.82)	1.00 (0.98 to 1.02)	.36	NS
Temporal	61.62 (56.49 to 68.33)	61.57 (57.51 to 68.64)	+0.58 (−0.51 to +1.61)	1.01 (0.99 to 1.03)	.10	NS
Parietal	66.93 (61.77 to 71.19)	67.36 (60.56 to 71.32)	0.00 (−1.17 to +0.69)	1.00 (0.98 to 1.01)	.80	NS
Occipital	42.83 (38.93 to 46.12)	43.76 (40.37 to 46.47)	+0.18 (−0.68 to +1.57)	1.01 (0.98 to 1.04)	.02	NS
Supratentorial gray matter special regions						
Basal ganglia	10.48 (9.03 to 11.47)	10.25 (9.16 to 11.26)	−0.06 (−0.32 to +0.23)	0.99 (0.97 to 1.02)	.63	NS
Hippocampal complex	8.96 (8.36 to 9.63)	8.90 (8.27 to 9.60)	+0.03 (−0.20 to +0.22)	1.00 (0.98 to 1.03)	.64	NS
Insula	7.19 (6.69 to 8.15)	7.30 (6.56 to 8.12)	−0.09 (−0.23 to +0.10)	0.99 (0.97 to 1.01)	.52	NS
Thalamus	7.89 (7.39 to 8.81)	7.78 (7.23 to 8.61)	−0.07 (−0.44 to +0.16)	0.99 (0.95 to 1.02)	.05	NS
Other	0.13 (0.09 to 0.15)	0.13 (0.11 to 0.16)	+0.01 (−0.01 to +0.03)	1.06 (0.91 to 1.28)	.02	NS

Abbreviations: FDR, false discovery rate; IQR, interquartile range; MR1, first magnetic resonance scan after injury; MR2, second magnetic resonance scan after injury; NS, not significant; ROI, region of interest.

^a^ Eighty-one patients with mild traumatic brain injury received a magnetic resonance scan within 72 hours of injury (MR1) and 2 to 3 weeks after injury (MR2).

^b^ Reported values are unadjusted.

^c^ Indicates which results are statistically significant using a 5% FDR threshold.

Although at MR1 these 3 ROIs did not differ significantly between patients and controls (eg, patient to control ratio for white matter volume, 0.99; 95% CI, 0.97-1.01; *P* = .24) (eTable 4 in the Supplement), at MR2, patients had significantly larger ventricles (patient to control ratio, 1.19; 95% CI, 1.08-1.32; *P* = .001) and smaller cerebral white matter volumes (patient to control ratio, 0.97; 95% CI, 0.95-0.99; *P* < .001). At MR3, patients’ white matter volumes had not changed significantly from MR2 (MR3:MR2 ratio, 1.01; IQR, 1.00-1.04 [95% CI, 0.99-1.04]; *P* = .17) and remained low compared with controls (patient to control ratio, 0.96; 95% CI, 0.94-0.99; *P* = .001).

### DTI Analysis

Diffusion data was available for 73 MR1 and 79 MR2 scans. Between MR1 and MR2, fractional anisotropy did not change significantly in any tract. Mean diffusivity changed significantly in 13 tracts, 12 of which displayed a decrease: the superior longitudinal fascicles I and II bilaterally, superior longitudinal fascicle III, arcuate fascicle, cingulum, middle longitudinal fascicle, thalamoprecentral tract, left striatoprecentral tract, and striatoparietal tract on the left and the right corticospinal tract. Mean diffusivity increased significantly in the left fornix.

Where patients differed significantly from controls, they had higher mean diffusivity and lower fractional anisotropy values. After correction for multiple comparisons, this imaging signature applied to the following: all assessed tracts except the left fornix (mean diffusivity at MR1); all except the right superior longitudinal fascicle I and right corpus callosum (mean diffusivity at MR2); and the left superior longitudinal fascicle I, left middle longitudinal fascicle, left striatoparietal tract, and corpus callosum (fractional anisotropy at MR1).

There was no significant within-patient difference between MR2 and MR3. However, the higher mean diffusivity values observed in patients compared with controls were no longer significant at MR3, possibly owing to the smaller number of patients available at MR3. Results of DTI for the individual 72 tracts at all points are available on request.

### DTI Trajectories Between MR1 and MR2

**Figure 1.**
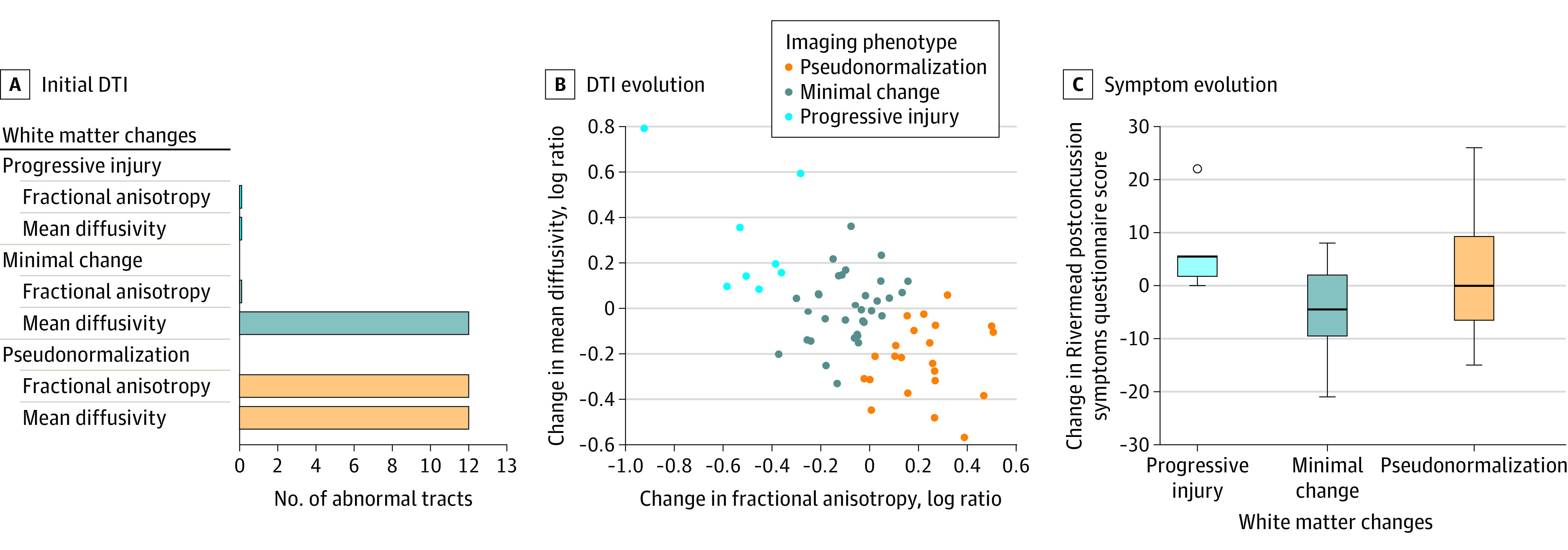
Association of Imaging Results With Symptom Evolution Between Magnetic Resonance Imaging (MRI) Times Patients with mild traumatic brain injury received an MRI scan within 72 hours of injury (MR1) and 2 to 3 weeks after injury (MR2). The evolution of diffusion tensor imaging (DTI) findings between scans was categorized into 3 phenotypes: progressive injury, minimal change, and pseudonormalization. A, DTI parameters at MR1 between patients with phenotypes and healthy controls, adjusted for age, sex, and scanner. The x-axis refers to the number of tracts with abnormal findings on MR1 of the short list of 13 tracts that were shown to change between MR1 and MR2. B, Evolution of DTI parameters between MR1 and MR2 (ie, the change in mean diffusivity vs the change in fractional anisotropy). Positive log ratios indicate an increase and negative log ratios a decrease in values between scans. C, Evolution of symptoms between scans measured as change in the score of the Rivermead Post Concussion Symptoms Questionnaire, with positive values indicating worsening and negative values resolving symptoms. The boxes represent the interquartile range, the middle horizontal line is the median, and the whiskers extend to the largest value no farther than 1.5 IQR from the hinge.

Sixty-three patients had diffusion data available for all 13 tracts that changed between MR1 and MR2. These data were used to derive 3 imaging phenotypes (Figure 1B): patients with decreasing mean diffusivity and increasing fractional anisotropy (pseudonormalization), those with little change in diffusion parameters (minimal change), and those with increasing mean diffusivity and decreasing fractional anisotropy (progressive injury). These are descriptive labels, not implying an underlying mechanism.

The pseudonormalization phenotype differed from controls in both mean diffusivity and fractional anisotropy at MR1 (12 of 13 and 12 of 13 tracts had abnormal findings, respectively) but normalized on MR2 (0 of 13 and 0 of 13 tracts had abnormal findings, respectively). In the minimal change phenotype, mean diffusivity but not fractional anisotropy differed from controls at MR1 (12 of 13 and 0 of 13 tracts had abnormal findings, respectively), and this diffusivity persisted at MR2 (6 of 13 and 0 of 13 tracts had abnormal findings, respectively). Interestingly, the progressive injury phenotype, despite showing within-patient deterioration of diffusion parameters, did not, as a group, differ significantly from controls at either scan.

### Symptom Trajectories Between MR1 and MR2

Baseline RPQ scores did not differ among image-based phenotypes (9.00 [IQR, 7.50-18.00] for pseudonormalization; 12.00 [IQR, 8.00-20.75] for minimal change; and 10.00 [IQR, 2.75-18.75] for progressive injury; *P* = .75). Symptom evolution, however, was significantly associated with phenotypes (Figure 1C), even after sensitivity analysis (eTable 5 in the Supplement). The RPQ scores deteriorated in the progressive injury phenotype (+5.00 [IQR, +2.00 to +5.00]), improved in the minimal change phenotype (−4.50 [IQR, −9.25 to +1.75]), and showed a variable evolution in the pseudonormalization phenotype (0.00 [IQR, −6.25 to +9.00]) (*P* = .02).

### Outcome Analysis

**Figure 2.**
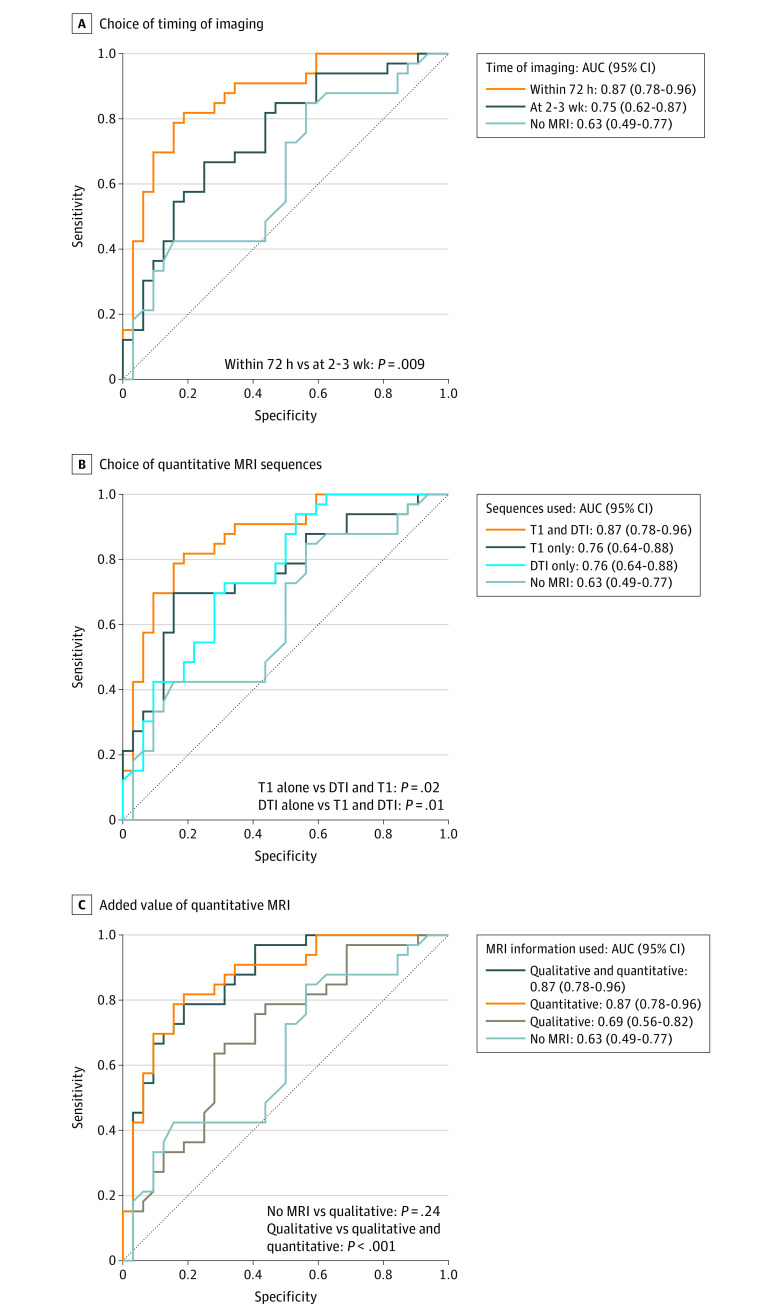
Estimation of Recovery at 3 Months Using Magnetic Resonance Imaging (MRI) Logistic regression was used to associate imaging with the odds of a favorable recovery at 3 months after injury, defined as a score on the extended Glasgow Outcome Scale of 8. The no-MRI model includes only age and sex. All other models contain age and sex plus imaging information. A, Models using imaging information obtained at 72 hours or 2 to 3 weeks after injury are compared. Imaging information includes both quantitative sequences (structural sequence [T1-weighted] and diffusion tensor imaging [DTI]). B, Imaging sequences obtained within 72 hours were compared. For T1, the variable used was the deviation of the patients’ cerebral white matter volume from that of healthy controls undergoing scanning on the same machine, whereby the volumes were normalized to each participant’s total intracranial volume. For DTI, variables included the number of tracts with abnormal findings with regard to fractional anisotropy, median diffusivity, or both compared with healthy controls undergoing scanning on the same machine. C, The added value of quantitative (T1-weighted and DTI) beyond qualitative information, that is, the presence or absence of any visible lesion reported by an expert who reviewed all available sequences (T1-weighted, T2-weighted, fluid-attenuated inversion recovery, and susceptibility-weighted imaging and DTI) is compared. AUC indicates area under the curve with 95% CI. *P* values were calculated using a paired DeLong test for comparing AUCs.

Recovery was favorable for 33 of 65 patients (51%) at 3 months. The association between recovery and imaging findings was significantly closer at MR1 than at MR2 (AUC, 0.87 [95% CI, 0.78-0.96] vs 0.75 [95% CI, 0.62-0.87; *P* = .009]; PPV, 0.79 vs 0.69; and NPV, 0.81 vs 0.67) (Figure 2A). Combining both sequences at MR1 (results above) was superior to using T1 weighting (AUC, 0.76 [95% CI, 0.64-0.88; *P* = .02]; PPV, 0.81; and NPV, 0.71) or DTI (AUC, 0.76 [95% CI, 0.64-0.88; *P* = .01]; PPV, 0.62; and NPV, 0.65) alone (Figure 2B). Quantitative imaging added value beyond the visible lesion presence (AUC, 0.87 [95% CI, 0.78-0.96] vs 0.69 [95% CI, 0.56-0.82; *P* < .001]; PPV, 0.72 vs 0.68; and NPV, 0.76 vs 0.65) (Figure 2C). All results were robust to sensitivity analyses (eTables 6 and 7 and eFigure 2 in the Supplement).

## Discussion

To our knowledge, this is the largest analysis to date of patients with mTBI undergoing serial MRI within the first few weeks after injury^44,45,46,47^ and the first of those analyses to include multiple centers. Our data document the dynamic evolution of both conventional and advanced MRI (using DTI), with the earlier point (<72 hours) showing potential prognostic value.

Regarding conventional MRI, a longitudinal study found traumatic lesions in 12% of patients with mTBI within 72 hours, half of which were consistent with traumatic axonal injury.^10^ Similar to our findings, traumatic axonal injury and subarachnoid hemorrhage remained visible on later scans; however, intraventricular hemorrhage was not reported. Although no intervention was needed for subarachnoid hemorrhage and intraventricular hemorrhage, these lesions have prognostic significance in moderate to severe TBI^17^ and may help select enriched populations for clinical follow-up or interventional trials also in mTBI.

The reduction in white matter volume between MR1 and MR2 could either be ascribed to resolution of early edema or to late loss of white matter. Compared with controls, patients had similar volumes at MR1 but reduced volumes at MR2. This finding suggests that the reduction of white matter volume at MR2 (which persisted at MR3) did not represent resolution of edema but rather new, persistent, and potentially progressive pathology (eg, Wallerian degeneration).^48^ Similarly, a study of 14 patients with mTBI^46^ found an enlargement of ventricles and cerebrospinal fluid volume between 72 hours and 1 month using voxel-based morphometry.

Limited literature is available on ultra-early DTI in mTBI. A longitudinal study of 25 patients detected widespread reductions in fractional anisotropy compared with controls at 72 hours and 3 months.^12^ In football players (n = 26) undergoing imaging 24 hours after a concussion, mean diffusivity was decreased compared with that of controls bilaterally in several regions and remained low at 8 days.^44^ Interestingly, within individuals, imaging findings did not change. Although a small study of 20 patients with mTBI detected within-patient changes in fractional anisotropy within that first week after injury,^47^ this outcome may suggest that markers of injury progression/resolution become visible closer to the 2- to 3-week point used in the present study. Supporting this notion, a study of 33 patients undergoing imaging within 7 days after injury found reductions in fractional anisotropy, which partially recovered at 1 and 3 months.^45^ Similarly, the aforementioned study of imaging in 14 patients with mTBI at 72 hours and 1 month reported a fall in mean diffusivity and a rise in fractional anisotropy between scans.^46^ Values remained abnormal compared with those of controls at both points.^46^ A comparison of various MRI modalities in 62 patients with mTBI within 24 hours of injury found that only DTI was sensitive enough to detect changes relative to orthopedic controls.^49^ We also found that abnormalities are demonstrable within 72 hours using DTI. Importantly, abnormalities are detectable even when conventional imaging results are normal. Although these abnormalities evolve within the first month of injury, they persist at 3 months.

In addition to replicating past results, we have, for the first time to our knowledge, parsed MRI and DTI changes in mTBI by deriving clinically plausible imaging phenotypes that were associated with symptom trajectories. Such diversity in the host response to injury may explain why past studies, which assumed a uniform response among all patients, do not always agree on the direction or magnitude of DTI changes. Previous studies considered diffusion parameters in isolation rather than the interaction of fractional anisotropy and mean diffusivity, which may further explain inconsistent results when trying to determine the association between DTI abnormalities and early mTBI symptoms.^45,47,50^ We noted that patients with progressive changes in their DTI metrics had worsening RPQ scores, which agrees with previous work in moderate to severe TBI.^13^

Perhaps most importantly, the earliest imaging time point (<72 hours) but less so the second (2-3 weeks) showed good prognostic value. A previous study^12^ (n = 25) found no difference on ultra-early DTI between patients with and without persistent postconcussional symptoms. That analysis, however, only examined individual tracts, whereas we derived a whole-brain measure of DTI derangement to better account for the heterogeneity of injury location. Consistent with our approach, a study in which 76 patients underwent imaging 5 to 18 days after injury found an association between outcome and having at least 1 abnormal white matter ROI (defined as fractional anisotropy >2.2 SDs below the control mean).^11^ Our data suggest that the earlier the MRI, the closer the association with outcome after mTBI. However, the pattern of white matter injury likely varies between individuals owing to both injury and host factors. Better detection of injury (eg, with multishell diffusion MRI), refined analysis with smaller ROIs, more complex models for outcome prediction (eg, using machine learning), and larger patient numbers may all refine the role of MRI in general and ultra-early MRI in particular.

### Strengths and Limitations

Strengths of this multicenter study include use of clinical scanners (not only research-dedicated ones), which provided a more generalizable assessment of the magnitude of changes detectable in clinical practice. This study also has some limitations. Although our sample size of 81 patients compares well with past serial studies of ultra-early imaging, future studies will need larger numbers to allow external validation of our findings before translation into clinical practice. Larger numbers would also allow for the inclusion of additional covariates in the outcome models, such as Marshall score, extracranial injury, or prior mental health, to better understand what MRI adds beyond currently available clinical information. Future studies may also benefit from extra time points to refine the optimal timing for MRI. In addition, more sensitive outcome measures are required to determine whether MRI could further differentiate between those patients who fully recovered and those with persistent, albeit nondisabling, symptoms (both extended Glasgow Outcome Scale scores of 8).

## Conclusions

In this cohort study, acute mTBI symptoms as well as longer-term functional outcome were associated with white matter changes detectable on advanced MRI. Imaging may thus document the evolution of pathology, thereby highlighting windows for therapy. In addition, imaging can provide prognostic information to help select patients for clinical follow-up or interventional trials. Importantly, our findings demonstrate that MRI appearances are dynamic and that images obtained closer to the time of injury are more strongly associated with outcome. Future studies with more patients and time points will help to further establish the optimal timing and clinical utility of MRI after mTBI.

## Notes

Supplement. **eFigure 1.** Flowchart of Patients Included in the Analysis **eTable 1.** Overview of Statistical Methods **eTable 2.** Comparison of Patients With and Without Data Available for Each Analysis **eTable 3.** Comparison of Lesions Visible on CT vs MR1 **eTable 4.** Comparison of Volumes Between Patients and Controls **eTable 5.** Sensitivity Analysis of Symptom Evolution **eTable 6.** Components of the Outcome Models **eTable 7.** Sensitivity Analysis of Outcome Models **eFigure 2.** Analysis With and Without Patients Who Have Mass Lesions on CT
