# Supplementary material for: Neuroanatomical Substrates and Symptoms Associated With Magnetic Resonance Imaging of Patients With Mild Traumatic Brain Injury
Source: JAMA Netw Open. 2021 Mar 18;4(3):e210994. doi: 10.1001/jamanetworkopen.2021.0994 (PMC7974642; doi:10.1001/jamanetworkopen.2021.0994)
Supplement: Supplement. — eFigure 1. Flowchart of Patients Included in the Analysis eTable 1. Overview of Statistical Methods eTable 2. Comparison of Patients With and Without Data Available for Each Analysis eTable 3. Comparison of Lesions Visible on CT vs MR1 eTable 4. Comparison of Volumes Between Patients and Controls eTable 5. Sensitivity Analysis of Symptom Evolution eTable 6. Components of the Outcome Models eTable 7. Sensitivity Analysis of Outcome Models eFigure 2. Analysis With and Without Patients Who Have Mass Lesions on CT [file jamanetwopen-e210994-s001.pdf]

Supplementary Online Content

Richter S, Winzeck S, Kornaropoulos EN, et al; Collaborative European NeuroTrauma Effectiveness Research in Traumatic Brain Injury Magnetic Resonance Imaging (CENTER-TBI MRI) Substudy Participants and Investigators. Neuroanatomical substrates and symptoms associated with magnetic resonance imaging of patients with mild traumatic brain injury. *JAMA Netw Open.* 2021;4(3):e210994. doi:10.1001/jamanetworkopen.2021.0994

eFigure 1. Flowchart of Patients Included in the Analysis

eTable 1. Overview of Statistical Methods

eTable 2. Comparison of Patients With and Without Data Available for Each Analysis

eTable 3. Comparison of Lesions Visible on CT vs MR1

eTable 4. Comparison of Volumes Between Patients and Controls

eTable 5. Sensitivity Analysis of Symptom Evolution

eTable 6. Components of the Outcome Models

eTable 7. Sensitivity Analysis of Outcome Models

eFigure 2. Analysis With and Without Patients Who Have Mass Lesions on CT

This supplementary material has been provided by the authors to give readers additional information about their work.

© 2021 Richter S et al. *JAMA Network Open*.

eFigure 1. Flowchart of Patients Included in the Analysis

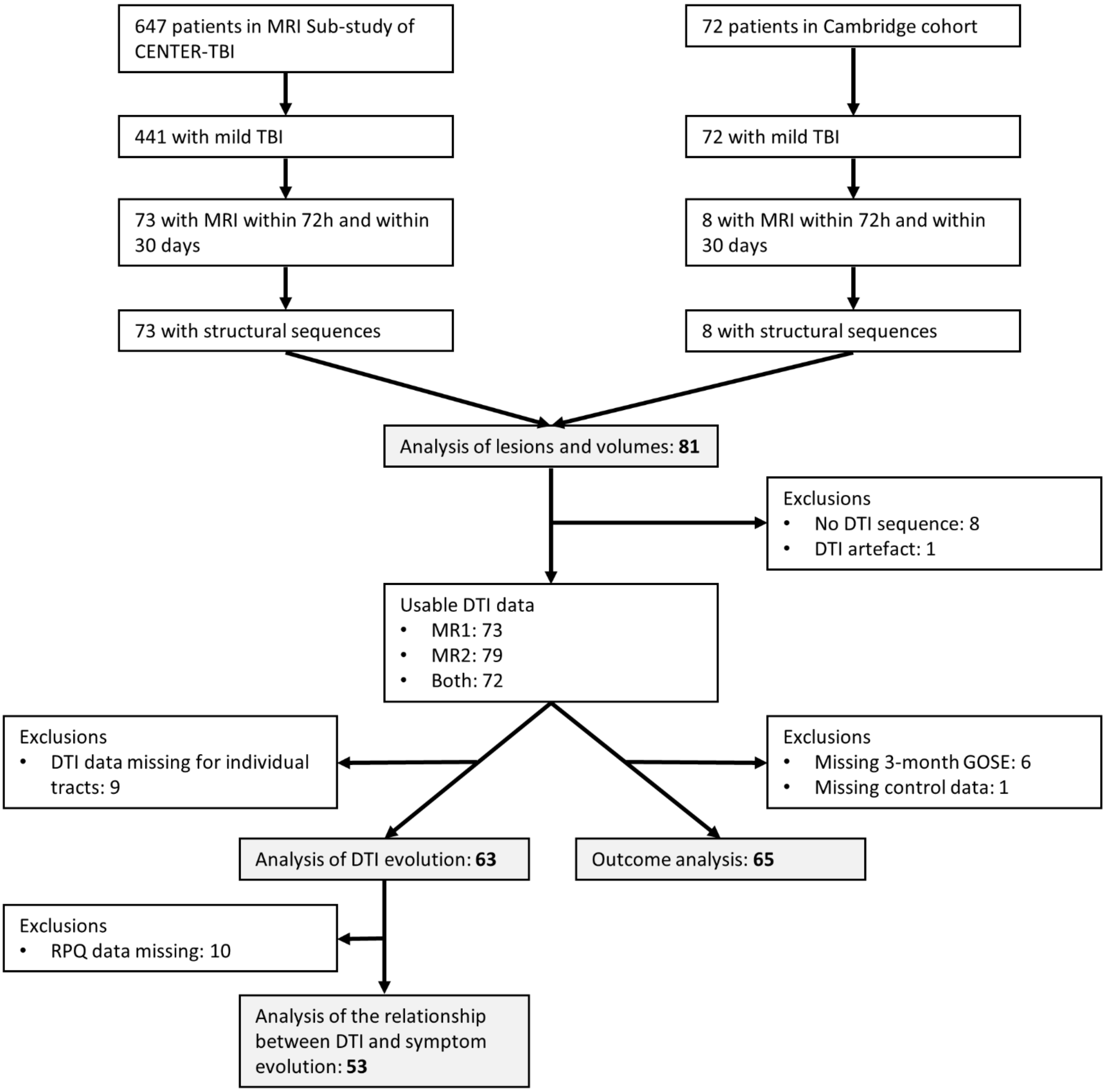

Structural MRI sequences included T1 weighted, T2 weighted, fluid-attenuated inversion recovery and susceptibility weighted imaging.

© 2021 Richter S et al. *JAMA Network Open*.

eTable 1. Overview of Statistical Methods

Question	Analysis type	Brain regions included	Statistical test	Detailed description
1. **Did the incidence of radiologically visible lesions differ between MR1 and MR2?**	univariate	All 10 lesion types	McNemar's test for paired categorical data	-
2. **Within patients, did the overall volumetric composition of brain ROIs change from MR1 to MR2?**	multi-variate	All 15 ROIs (grey and white matter)	Two-tailed Hotelling's one-sample T2 test	We performed a compositional data analysis as described by Aitchinson. In short, first the within-patient change of each ROI relative to the patient's intracranial volume ("perturbation") is calculated. To account for the correlation between ROIs, all perturbations for each patient were transformed using an additive log ratio (alr) resulting in one alr vector per patient. To decide whether there is a compositional change between MR1 and MR2, we tested whether the mean of all alr vectors differed from zero using the Hotelling's one-sample T^2 test for multivariate data. Note that since serial scans of the same patient were always performed on the same scanner, there were no scanner differences to adjust for.
3. **Within patients, did individual ROIs change in volume between MR1 and MR2 (or MR2 and MR3)?**	univariate	All 15 ROIs (grey and white matter)	two-tailed one-sample t-test	For each ROI, the within-patient change was summarised in a single value as log(Volume on MR2/Volume on MR1) negating the need for a two-sample or paired t-test. The one-sample t-test assessed whether the mean change of all patients differed significantly from zero. The transformation into a log-ratio also ensured that data was normally distributed. Note that since serial scans of the same patient were always performed on the same scanner, there were no scanner differences to adjust for.
4. **Did ROI volumes differ between patients and controls at MR1 (or at MR2 or at MR3)?**	univariate	Those 3 ROIs that changed between MR1 and MR2	mixed model	ROI volume was first normalised for each person's total intracranial volume (by taking the ratio ROI/total intracranial volume) and then modelled as follows: Log(Volume) ~ group + age + sex + (1|scanner), where "group" categorised each person as either patient or control. We tested if "group" was significant according to p-values generated via Satterthwaite's degrees of freedom method (package lmerTest 3.1-2). Note that "scanner" refers to individual machines, not just scanner models, so that there are no residual site effects even if two sites used the same model. Assumptions were tested using diagnostic plots.
5. **Did FA (or MD) change in individual tracts between MR1 and MR2?**	univariate	All 72 white matter tracts	Two-tailed one sample t-test	For each tract, the within-patient change was summarised in a single value as log(FA on MR2/FA on MR1) negating the need for a two-sample or paired t-test. This transformation into a logratio also ensured that data was normally distributed. The analogous method was used for MD.
6. **Did individual tracts differ from controls in their FA (or MD) values?**	univariate	Those 13 tracts that changed between MR1 and MR2 plus	mixed model	FA ~ group + age + sex + (1|scanner), where "group" categorised each person as either patient or control. We tested if "group" was significant according to p-values generated via Satterthwaite's degrees of freedom method (package lmerTest 3.1-2). The analogous method was used for MD. Note that "scanner" refers to individual machines, not just

©2021 Richter S et al. *JAMA Network Open*.

		corpus callosum		scanner models, so that there are no residual site effects even if two sites used the same model. Assumptions were tested using diagnostic plots.
7. **Which of the three phenotypes best describes the DTI changes between MR1 and MR2 for each patient?**	bivariate	Those 13 tracts that changed between MR1 and MR2	k-means clustering	For each tract, the within-patient change was summarised in a single value as log(FA on MR2/FA on MR1). For each patient these 13 single values were added to provide a summary measure of FA change in that patient. The analogous method was used for MD. Clusters were based on two variables: the summary FA change and the summary MD change. The log-ratio transformation ensured both variables are on the same scale.
8. **How did the three phenotypes differ from controls at MR1 (or MR2)?**	univariate	Those 13 tracts that changed between MR1 and MR2	mixed model	We defined the first model as FA ~ group + age + sex + (1|scanner), where "group" categorised each person as either control, pseudonormalisation-phenotype, minimal change-phenotype or progressive injury-phenotype. To decide if "group" had a significant effect we compared the first model with FA ~ age + sex + (1|scanner) using the Chi-squared test. Since "group" had a significant effect for all tracts, p-values were generated via Satterthwaite's degrees of freedom method (package lmerTest 3.1-2) for the coefficients of each phenotype. For each phenotype p-values were corrected using a false discovery rate threshold of 5% and the number of tracts counted that significantly differed from controls. Assumptions were tested using diagnostic plots.
9. **Did initial mTBI symptoms at the time of MR1 differ between phenotypes?**	univariate	n/a	ANOVA	Continuous y-variable: RPQ scores at MR1, Categorical x-variable: phenotype. Assumptions were tested using diagnostic plots.
10. **Did symptoms progress differently in different phenotypes?**	univariate	n/a	ANOVA	Continuous y-variable: ΔRPQ score (i.e. RPQ at MR2 minus RPQ at MR1), Categorical x-variable: phenotype. Assumptions were tested using diagnostic plots.
11. **Is imaging associated with clinical outcome?**	univariate	All 72 white matter tracts	logistic regression	The y-variable was binary: favorable recovery (GOSE = 8) vs. unfavorable recovery (GOSE < 8). The x-variables included age (continuous), sex (binary) and, where appropriate,"lesion presence" obtained from structured radiology reports, "WM volume" obtained from T1 imaging, and "fa tracts", "md tracts" and "both tracts" from DTI imaging. "Lesion presence" is a binary variable indicating the presence or absence of any visible lesion on any available sequence. "WM volume" is a continuous variable describing by how many standard deviations the patient's cerebral WM volume (normalised for their total brain volume) deviated from the mean of controls scanned on the same machine. The DTI variables where nominal and counted how many of the 72 tracts in each patient where abnormal with respect to only FA, only MD or both. Abnormal meant >2SD below (for FA) or above (for MD) the control mean. This binary classification resulted in better model performance than classifying FA (or MD) as high/normal/low and allowed the inclusion of the variable "both tracts" without resulting in multicollinearity as measured by the generalized variance-inflation factor corrected by the number of degrees of freedom

©2021 Richter S et al. *JAMA Network Open*.

				GVIF^(1/(2*Df)) with a threshold of 2 (analogous of VIF = 4). Model assumptions were tested using diagnostic pots, the Box-Tidwell test and the GVIF.
**12. Which imaging timepoint and sequences is more closely associated with outcome?**	n/a	n/a	AUC, CV, AIC	The above logistic regression models where compared using three measures: the area under the receiver operating characteristic curve (AUC), ten-fold cross-validation (CV) and the Akaike information criterion (AIC). To obtain the AUC, observed and predicted outcome was compared for all patients with available data. Two AUCs were compared using a paired DeLong's test. The CV accuracy is the average accuracy of ten measures obtained by randomly splitting the data into ten folds and repeatedly training the model on nine folds and testing it on the remaining fold. When comparing two models based on AIC, we considered a model to fit the data significantly better if its AIC was at least 2 units lower than that of the alternative model.
**13. Are conclusions from Q10-12 robust even though patients with missing outcome data were excluded from the analyses?**	n/a	n/a	Sensitivity analysis (best- and worst-case scenario)	Sensitivity analysis for Q10: Some patients had been excluded from the complete-case analysis as they were missing ΔRPQ data. For the worst-case scenario, we assumed all 10 patients had deteriorated and imputed a ΔRPQ of +5 (the median observed in the progressive injury phenotype). For the best-case scenario, we assumed all 10 patients had improved and imputed a ΔRPQ of -4.5 (the median observed in the minimal change phenotype). An ANOVA as per Q10 was then conducted for both scenarios.<br><br>Sensitivity analysis for Q11-12: Some patients were excluded from the predictions models as they were missing GOSE data. For these patients an incomplete recovery was imputed for the worst-case scenario and a complete recovery for the best-case scenario. Logistic regression and an assessment of model performance was then conducted as per Q11 and Q12.

MR1/MR2/MR3 = Magnetic resonance scan performed within 72h/at 2–3 weeks/at 3-months after injury, ROI = Region of interest, FA = fractional anisotropy, MD = mean diffusivity, WM = white matter, SD = standard deviation. Statistical significance was determined by applying a false discovery rate threshold of 5% within each question.

©2021 Richter S et al. *JAMA Network Open*.

eTable 2. Comparison of Patients With and Without Data Available for Each Analysis

Analysis	Structural	DTI evolution between scans			Symptom evolution between scans			Outcome analysis		
	Overall (n=81)	Included (n=63)	Excluded (n=18)	Raw p-value	Included (n=53)	Excluded (n=28)	Raw p-value	Included (n=65)	Excluded (n=16)	Raw p-value
**Age**										
Median (Q1-Q3)	45 (24 - 59)	47 (27.5 - 59)	36 (22 - 56.8)	0.30	46 (23 - 59)	42 (28 - 56.2)	0.77	47 (25 - 59)	36 (20.5 - 56.2)	0.22
**Sex**										
F	24 (30 %)	18 (29 %)	6 (33 %)	0.77	16 (30 %)	8 (29 %)	1.00	18 (28 %)	6 (38 %)	0.54
M	57 (70 %)	45 (71 %)	12 (67 %)		37 (70 %)	20 (71 %)		47 (72 %)	10 (62 %)	
**Education**										
Completed degree	33 (41 %)	29 (46 %)	4 (22 %)	0.09	24 (45 %)	9 (32 %)	0.17	28 (43 %)	5 (31 %)	0.53
Current degree	1 (1 %)	0 (0 %)	1 (6 %)		0 (0 %)	1 (4 %)		1 (2 %)	0 (0 %)	
High school	25 (31 %)	20 (32 %)	5 (28 %)		16 (30 %)	9 (32 %)		21 (32 %)	4 (25 %)	
Post-high school trained	11 (14 %)	10 (16 %)	1 (6 %)		10 (19 %)	1 (4 %)		9 (14 %)	2 (12 %)	
Primary school	4 (5 %)	2 (3 %)	2 (11 %)		2 (4 %)	2 (7 %)		2 (3 %)	2 (12 %)	
Missing	7 (8.6%)	2 (3.2%)	5 (27.8%)		1 (1.9%)	6 (21.4%)		4 (6.2%)	3 (18.8%)	
**Mechanism of Injury**										
Acc-/deceleration	10 (12 %)	7 (11 %)	3 (17 %)	0.75	6 (11 %)	4 (14 %)	0.62	8 (12 %)	2 (12 %)	0.80
Blow to head	7 (9 %)	7 (11 %)	0 (0 %)		6 (11 %)	1 (4 %)		7 (11 %)	0 (0 %)	
Fall from height	21 (26 %)	15 (24 %)	6 (33 %)		11 (21 %)	10 (36 %)		16 (25 %)	5 (31 %)	
Ground level fall	19 (23 %)	15 (24 %)	4 (22 %)		13 (25 %)	6 (21 %)		16 (25 %)	3 (19 %)	
Head against object	11 (14 %)	9 (14 %)	2 (11 %)		7 (13 %)	4 (14 %)		8 (12 %)	3 (19 %)	
Multimechanistic	13 (16 %)	10 (16 %)	3 (17 %)		10 (19 %)	3 (11 %)		10 (15 %)	3 (19 %)	
**GCS**										
Median (Q1-Q3)	15 (15 - 15)	15 (15 - 15)	15 (14.2 - 15)	0.39	15 (15 - 15)	15 (14 - 15)	0.20	15 (15 - 15)	15 (14 - 15)	0.07
**ISS**										
Median (Q1-Q3)	8.5 (4 - 16.2)	8.0 (4 - 10)	17 (9 - 27)	0.005	5.0 (4 - 9)	20 (9 - 28)	<0.001	8.0 (4 - 10)	17 (9 - 27)	0.02
Missing	1 (1.2%)	0 (0%)	1 (5.6%)		0 (0%)	1 (3.6%)		0 (0%)	1 (6.2%)	
**Stratum**										
ER	42 (52 %)	37 (59 %)	5 (28 %)	0.06	36 (68 %)	6 (21 %)	<0.001	36 (55 %)	6 (38 %)	0.24
Admission	30 (37 %)	20 (32 %)	10 (56 %)		17 (32 %)	13 (46 %)		21 (32 %)	9 (56 %)	
ICU	9 (11 %)	6 (10 %)	3 (17 %)		0 (0 %)	9 (32 %)		8 (12 %)	1 (6 %)	

©2021 Richter S et al. *JAMA Network Open*.

**Marshall score**										
1	57 (70 %)	45 (71 %)	12 (67 %)	0.85	41 (77 %)	16 (57 %)	0.11	44 (68 %)	13 (81 %)	0.71
2	18 (22 %)	13 (21 %)	5 (28 %)		10 (19 %)	8 (29 %)		16 (25 %)	2 (12 %)	
3	0 (0 %)	0 (0 %)	0 (0 %)		0 (0 %)	0 (0 %)		0 (0 %)	0 (0 %)	
4	0 (0 %)	0 (0 %)	0 (0 %)		0 (0 %)	0 (0 %)		0 (0 %)	0 (0 %)	
5	1 (1 %)	1 (2 %)	0 (0 %)		0 (0 %)	1 (4 %)		1 (2 %)	0 (0 %)	
6	5 (6 %)	4 (6 %)	1 (6 %)		2 (4 %)	3 (11 %)		4 (6 %)	1 (6 %)	
**CWM ratio**										
Median (Q1-Q3)	0.98 (0.96 - 1)	0.99 (0.96 - 1)	0.98 (0.97 - 0.99)	0.61	0.99 (0.96 - 1)	0.98 (0.97 - 0.99)	0.60	0.98 (0.96 - 1)	0.99 (0.97 - 0.99)	0.70
**Phenotype**										
Minimal change	33 (41 %)	33 (52 %)	0 (0 %)	1.00	30 (57 %)	3 (11 %)	0.09	29 (45 %)	4 (25 %)	0.18
Progressive injury	8 (10 %)	8 (13 %)	0 (0 %)		5 (9 %)	3 (11 %)		8 (12 %)	0 (0 %)	
Pseudonormalisation	22 (27 %)	22 (35 %)	0 (0 %)		18 (34 %)	4 (14 %)		22 (34 %)	0 (0 %)	
Missing	18 (22.2%)	0 (0%)	18 (100%)		0 (0%)	18 (64.3%)		6 (9.2%)	12 (75.0%)	
**ΔRPQ**										
Median (Q1-Q3)	-1.0 (-7 - 5)	-2.0 (-7 - 5)	-1.0 (-1 - 3)	0.46	-2.0 (-7 - 5)	-1.0 (-1 - 3)	0.46	0.0 (-7 - 5)	-1.0 (-5.2 - 0.5)	0.77
Missing	24 (29.6%)	10 (15.9%)	14 (77.8%)		0 (0%)	24 (85.7%)		16 (24.6%)	8 (50.0%)	
**GOSE**										
Median (Q1-Q3)	7.0 (6.5 - 8)	8.0 (7 - 8)	7.0 (6 - 7.2)	0.07	8.0 (7 - 8)	7.0 (6 - 8)	0.04	8.0 (7 - 8)	6.5 (6 - 7.8)	0.30
Missing	10 (12.3%)	4 (6.3%)	6 (33.3%)		4 (7.5%)	6 (21.4%)		0 (0%)	10 (62.5%)	
**MR1**										
Median (Q1-Q3)	36 (24.7 - 55.2)	43 (24.2 - 59)	31 (25 - 42)	0.19	40 (23.8 - 59.5)	34 (25.4 - 49.7)	0.67	44 (25 - 58.6)	30 (22.6 - 33.2)	0.03
**MR2**										
Median (Q1-Q3)	17 (14.8 - 21.1)	16 (14.7 - 20.8)	20 (18.3 - 21.7)	0.02	16 (14.6 - 20.5)	19 (16.3 - 21.8)	0.02	16 (14.8 - 21.2)	19 (16.3 - 20.4)	0.57
**MR3**										
Median (Q1-Q3)	97 (92 - 100)	96 (91.2 - 99.9)	99 (95.6 - 100)	0.75	96 (91.1 - 99.7)	99 (96.7 - 100.3)	0.50	97 (91.3 - 99.8)	97 (94.7 - 99.1)	0.92
Missing	42 (51.9%)	27 (42.9%)	15 (83.3%)		19 (35.8%)	23 (82.1%)		28 (43.1%)	14 (87.5%)	

©2021 Richter S et al. *JAMA Network Open*.

Data were compared using the Mann-Whitney test for numeric and Fisher's exact test for categorical data. Reported p-values are unadjusted. Applying a false discovery threshold of 5%, only p-values < 0.001 remained significant and are highlighted in grey. Thus, patients without RPQ data were more severely injured and more commonly required intensive care but had comparable GCS and Marshall scores, suggesting they were untestable due to extra-cranial injuries. Otherwise there was no significant difference between patients with and without available data. "Stratum" indicates whether the patient was discharged from the emergency department (ER), was admitted for standard care (Admission) or intensive care (ICU). GCS = Glasgow Coma Scale score on presentation, ISS = Injury Severity Score on presentation, CWM ratio = ratio of the volume of cerebral white matter on MR2/MR1, Phenotype = pattern of change in DTI parameters between scans, ΔRPQ = difference in Rivermead post-concussion symptoms questionnaire scores at MR2 minus MR1, GOSE = Glasgow Outcome Scale Extended, MR1/MR2/MR3 = serial magnetic resonance scans within 72h/at 2–3 weeks/at 3 months after injury.

©2021 Richter S et al. *JAMA Network Open*.

eTable 3. Comparison of Lesions Visible on CT vs MR1

	CT positive			CT negative			CT vs. MR1	
**Abnormality**	**CT positive**	**CT lesion also seen on MR1**	**CT lesion not seen on MR1**	**CT negative**	**MR1 also negative**	**MR1 lesion not seen on CT**	**Raw p-value**	**FDR**
Any Abnormality	24 (100%)	18 (75%)	6 (25%)	52 (100%)	39 (75%)	13 (25%)	0.17	
**Mass effect**								
Mass > 25cc	5 (100%)	2 (40%)	3 (60%)	71 (100%)	71 (100%)	0 (0%)	0.25	
Midline shift	2 (100%)	1 (50%)	1 (50%)	74 (100%)	74 (100%)	0 (0%)	1.00	
Cisternal compression	2 (100%)	1 (50%)	1 (50%)	74 (100%)	74 (100%)	0 (0%)	1.00	
**Intra-axial**								
Contusion	9 (100%)	9 (100%)	0 (0%)	67 (100%)	59 (88%)	8 (12%)	0.01	sig.
Traumatic axonal injury	0 (100%)	0	0	76 (100%)	57 (75%)	19 (25%)	<0.001	sig.
**Extra-axial**								
Epidural haemorrhage	4 (100%)	3 (75%)	1 (25%)	72 (100%)	72 (100%)	0 (0%)	1.00	
Subdural haemorrhage	7 (100%)	3 (43%)	4 (57%)	69 (100%)	65 (94%)	4 (6%)	1.00	
Subarachnoid haemorrhage	12 (100%)	7 (58%)	5 (42%)	64 (100%)	59 (92%)	5 (8%)	1.00	
**Other**								
Skull fracture	15 (100%)	0 (0%)	15 (100%)	61 (100%)	61 (100%)	0 (0%)	<0.001	sig.
Intraventricular haemorrhage	3 (100%)	2 (67%)	1 (33%)	73 (100%)	67 (92%)	6 (8%)	0.13	

81 patients received a computed tomography scan (CT) within 24h and a magnetic resonance scan (MR1) within 72h of their mild traumatic brain injury. The presence of visible lesions was compared between scans using McNemar’s test for categorical data. The column FDR indicates which p-values are statistically significant (sig.) based on a false discovery rate threshold of 5%.

©2021 Richter S et al. *JAMA Network Open*.

eTable 4. Comparison of Volumes Between Patients and Controls

Timepoint	ROI	Raw Coefficient	Standard Error	Ratio patient/ control	Unadjusted p-value	FDR-adjusted significance
MR1	Ventricles	0.069	0.054	1.07	0.20	not sig.
	Convexity CSF	-0.045	0.042	0.96	0.30	not sig.
	Cerebral white matter	-0.011	0.009	0.99	0.24	not sig.
MR2	Ventricles	0.176	0.053	1.19	0.001	significant
	Convexity CSF	0.039	0.039	1.04	0.31	not sig.
	Cerebral white matter	-0.032	0.008	0.97	0.00	significant

81 patients with mild traumatic brain injury were compared to healthy controls using mixed models, adjusted for age, sex, scanner and total intracranial volume. Magnetic resonance images were obtained within 72h (MR1) and at 2–3 weeks (MR2) after injury. FDR = false discovery rate with a 5% threshold.

©2021 Richter S et al. *JAMA Network Open*.

eTable 5. Sensitivity Analysis of Symptom Evolution

	ΔRPQ score, median [Q1-Q3]		
**Phenotype**	**Complete case analysis**	**Worst-case scenario**	**Best-case scenario**
Progressive injury	5.00 [2.00-5.00]	5.00 [4.25-5.00]	1.00 [-4.50-5.00]
Minimal change	-4.50 [-9.25-1.75]	-3.00 [-7.00-5.00]	-4.50 [-7.00-1.00]
Pseudonormalisation	0.00 [-6.25-9.00]	3.00 [-4.00-8.25]	-3.00 [-4.50-8.25]
P-value	0.02	0.008	0.05

Phenotype refers to the imaging phenotype i.e. the way in which diffusion parameters changed between 72h and 2-3 weeks after injury. ΔRPQ score = difference in Rivermead post-concussion symptoms questionnaire scores between 72h and 2-3 weeks, whereby a positive number indicates worsening symptoms and a negative number a reduction in symptoms. Complete case analysis excluded 10 patients for missing ΔRPQ data. The worst case scenario assumed mTBI symptoms in all 10 patients deteriorated, the best case scenario assumed all 10 patients improved. The table shows that even if RPQ data was missing not at random, the association between phenotypes and symptoms persists.

eTable 6. Components of the Outcome Models

Timepoint/ Sequences		Variable	Odds ratio	95% CI	P-value
No imaging		Intercept	3.26	0.86 - 13.7	0.09
		Age	0.97	0.94 – 1.00	0.04
		Female sex	1.94	0.63 - 6.39	0.26
MR1	T1	Intercepts	1.73	0.35 - 8.93	0.50
		Age	0.98	0.95 - 1.01	0.26
		Female sex	1.27	0.36 - 4.49	0.71
		**White matter volume**	**0.67**	**0.50 - 0.86**	**0.005**
	DTI	Intercept	9.8	1.81 - 69.78	0.01
		**Age**	**0.95**	**0.91 - 0.99**	**0.02**
		Female sex	1.31	0.35 - 5.2	0.69
		**Tracts with only FA abnormal**	**0.80**	**0.63 - 0.93**	**0.03**
		Tracts with only MD abnormal	1.00	0.95 - 1.06	0.88
		Tracts with both FA and MD abnormal	1.06	1.00 - 1.13	0.07
	T1 & DTI	Intercept	6.86	0.9 - 66.44	0.07
		Age	0.96	0.92 - 1.01	0.12
		Female sex	0.64	0.13 - 3.04	0.58
		**Tracts with only FA abnormal**	**0.79**	**0.58 - 0.92**	**0.04**
		Tracts with only MD abnormal	0.99	0.93 - 1.05	0.74
		**Tracts with both FA and MD abnormal**	**1.07**	**1.01 - 1.15**	**0.05**
		**White matter volume**	**0.60**	**0.4 - 0.82**	**0.004**
MR2	T1	Intercept	1.90	0.39 - 9.6	0.43
		Age	0.98	0.95 - 1.01	0.18
		Female sex	1.54	0.47 - 5.21	0.48
		White matter volume	0.86	0.67 - 1.07	0.18
	DTI	Intercept	6.07	1.23 - 36.25	0.03
		**Age**	**0.96**	**0.92 - 0.99**	**0.03**
		Female sex	1.78	0.5 - 6.84	0.38
		Tracts with only FA abnormal	0.91	0.80 – 1.00	0.08
		Tracts with only MD abnormal	1.01	0.95 - 1.07	0.79
		Tracts with both FA and MD abnormal	1.02	0.97 - 1.08	0.37
	T1 & DTI	Intercept	3.71	0.64 - 24.49	0.15
		Age	0.97	0.93 - 1.01	0.10
		Female sex	1.37	0.35 - 5.54	0.65
		**Tracts with only FA abnormal**	**0.90**	**0.79 - 0.99**	**0.06**
		Tracts with only MD abnormal	1.00	0.94 - 1.06	0.99
		Tracts with both FA and MD abnormal	1.03	0.98 - 1.08	0.31
		White matter volume	0.83	0.64 - 1.05	0.13
MR1	Qualitative only	Intercept	3.52	0.85 - 16.5	0.09
		Age	0.98	0.95 - 1.01	0.14
		Female sex	1.89	0.59 - 6.54	0.29
		Visible lesion present	0.39	0.13 - 1.11	0.08
	T1, DTI & Qualitative	Intercept	7.04	0.93 - 67.54	0.07
		Age	0.97	0.92 - 1.01	0.16
		Female sex	0.72	0.14 - 3.54	0.68
		Tracts with only FA abnormal	0.79	0.57 - 0.93	0.05
		Tracts with only MD abnormal	0.99	0.93 - 1.05	0.87
		**Tracts with both FA and MD abnormal**	**1.07**	**1.01 - 1.15**	**0.04**

©2021 Richter S et al. *JAMA Network Open*.

Timepoint/ Sequences		Variable	Odds ratio	95% CI	P-value
		**White matter volume**	**0.62**	**0.42 - 0.84**	**0.005**
		Visible lesion present	0.44	0.10 - 1.83	0.26

Logistic regression was used to identify the association between imaging and the odds of a favorable recovery at three months post-injury, defined as a score on the Glasgow Outcome Scale Extended of no less than 8. MR1 = magnetic resonance imaging obtained within 72h of injury. MR2 = magnetic resonance obtained 2-3 weeks post-injury. White matter volume = the deviation of the patients' cerebral white matter volume from that of healthy controls scanned on the same machine, whereby the volumes were normalised to each subject's total intracranial volume. DTI = Diffusion tensor imaging. Qualitative = the presence or absence of any visible lesion reported by an expert who reviewed all available sequences (T1 weighted, T2 weighted, fluid-attenuated inversion recovery, susceptibility weighted imaging and DTI). 95% CI = 95% Confidence Interval. In bold are Confidence intervals that do not include 1. P-values are unadjusted. Note that after correction for a false discovery threshold of 5% none of the p-values remain significant.

©2021 Richter S et al. *JAMA Network Open*.

eTable 7. Sensitivity Analysis of Outcome Models

		Complete case analysis					Worst-case scenario					Best-case scenario				
**Timepoint and Sequences**		**AUC (95% CI)**	**CV (95% CI)**	**AIC**	**PPV**	**NPV**	**AUC (95% CI)**	**CV (95% CI)**	**AIC**	**PPV**	**NPV**	**AUC (95% CI)**	**CV (95% CI)**	**AIC**	**PPV**	**NPV**
No imaging		0.65 (0.51-0.78)	0.50 (0.20-0.81)	94	0.53	0.53	0.63 (0.50-0.75)	0.60 (0.33-0.87)	108	0.62	0.65	0.64 (0.52-0.77)	0.58 (0.11-1.00)	107	0.64	0.57
MR1	T1 only	0.76 (0.64-0.88)	0.67 (0.35-1.00)	83	0.81	0.71	0.76 (0.64-0.87)	0.68 (0.41-0.95)	91	0.76	0.70	0.72 (0.6-0.84)	0.59 (0.26-0.91)	94	0.67	0.58
	DTI only	0.76 (0.64-0.88)	0.63 (0.41-0.85)	84	0.62	0.65	0.73 (0.61-0.84)	0.58 (0.33-0.84)	95	0.57	0.65	0.74 (0.63-0.86)	0.63 (0.37-0.89)	93	0.68	0.68
	T1 & DTI	0.87 (0.78-0.96)	0.73 (0.38-1.00)	74	0.79	0.81	0.86 (0.77-0.94)	0.71 (0.51-0.91)	83	0.74	0.79	0.84 (0.75-0.93)	0.71 (0.33-1.00)	85	0.74	0.77
MR2	T1 only	0.67 (0.53-0.8)	0.57 (0.32-0.82)	91	0.60	0.55	0.67 (0.54-0.79)	0.59 (0.28-0.9)	100	0.59	0.59	0.65 (0.52-0.78)	0.50 (0.11-0.89)	99	0.64	0.59
	DTI only	0.71 (0.58-0.84)	0.59 (0.23-0.96)	92	0.66	0.64	0.69 (0.56-0.81)	0.55 (0.23-0.86)	101	0.58	0.62	0.71 (0.58-0.83)	0.60 (0.22-0.97)	99	0.67	0.62
	T1 & DTI	0.75 (0.62-0.87)	0.58 (0.21-0.94)	92	0.69	0.67	0.73 (0.61-0.85)	0.61 (0.39-0.82)	100	0.69	0.69	0.73 (0.61-0.85)	0.59 (0.20-0.99)	100	0.67	0.68
MR1	Qualitative only	0.69 (0.56-0.82)	0.6 (0.09-1.00)	90	0.68	0.65	0.67 (0.55-0.80)	0.61 (0.30-0.92)	101	0.61	0.64	0.69 (0.57-0.82)	0.57 (0.14-1.00)	98	0.68	0.64
	T1, DTI & Qualitative	0.87 (0.78-0.96)	0.72 (0.37-1.00)	74	0.72	0.76	0.85 (0.77-0.94)	0.71 (0.47-0.95)	84	0.76	0.79	0.84 (0.75-0.93)	0.71 (0.34-1.00)	86	0.76	0.73

©2021 Richter S et al. *JAMA Network Open*.

Logistic regression was used to identify the association between imaging and the odds of a favourable recovery at three months post-injury, defined as a score of no less than 8 on the Glasgow Outcome Scale Extended. For the worst-case scenario, patients with missing outcome data were assumed to have had an unfavourable recovery. For the best-case scenario, a favourable recovery was assumed. The “no imaging” model includes only age and sex. All other models contain age and sex plus imaging information. MR1 = magnetic resonance imaging obtained within 72h of injury. MR2 = magnetic resonance obtained 2–3 weeks post-injury. T1 = the variable used was the deviation of the patients’ cerebral white matter volume from that of healthy control scanned on the same machine, whereby the volumes were normalised to each subject’s total intracranial volume. DTI = Diffusion tensor imaging; variables included the number of abnormal tracts with regards to fractional anisotropy, median diffusivity or both compared to healthy controls scanned on the same machine. Qualitative = the presence or absence of any visible lesion reported by an expert who reviewed all available sequences (T1 weighted, T2 weighted, fluid-attenuated inversion recovery, susceptibility weighted imaging and DTI). AUC = Area under the receiver operating characteristic curve. CV = Accuracy of predictive performance obtained from ten-fold cross-validation. 95% CI = 95% confidence interval. AIC = Akaike information criterion, note that lower values indicate better model fit. PPV = Positive predictive value. NPV = Negative predictive value. The table shows that, even if outcome data was missing not at random, MR1 continues to correlate more closely with outcome than MR2, and combining T1 and DTI sequences yields better results than using either sequence alone.

©2021 Richter S et al. *JAMA Network Open*.

eFigure 2. Analysis With and Without Patients Who Have Mass Lesions on CT

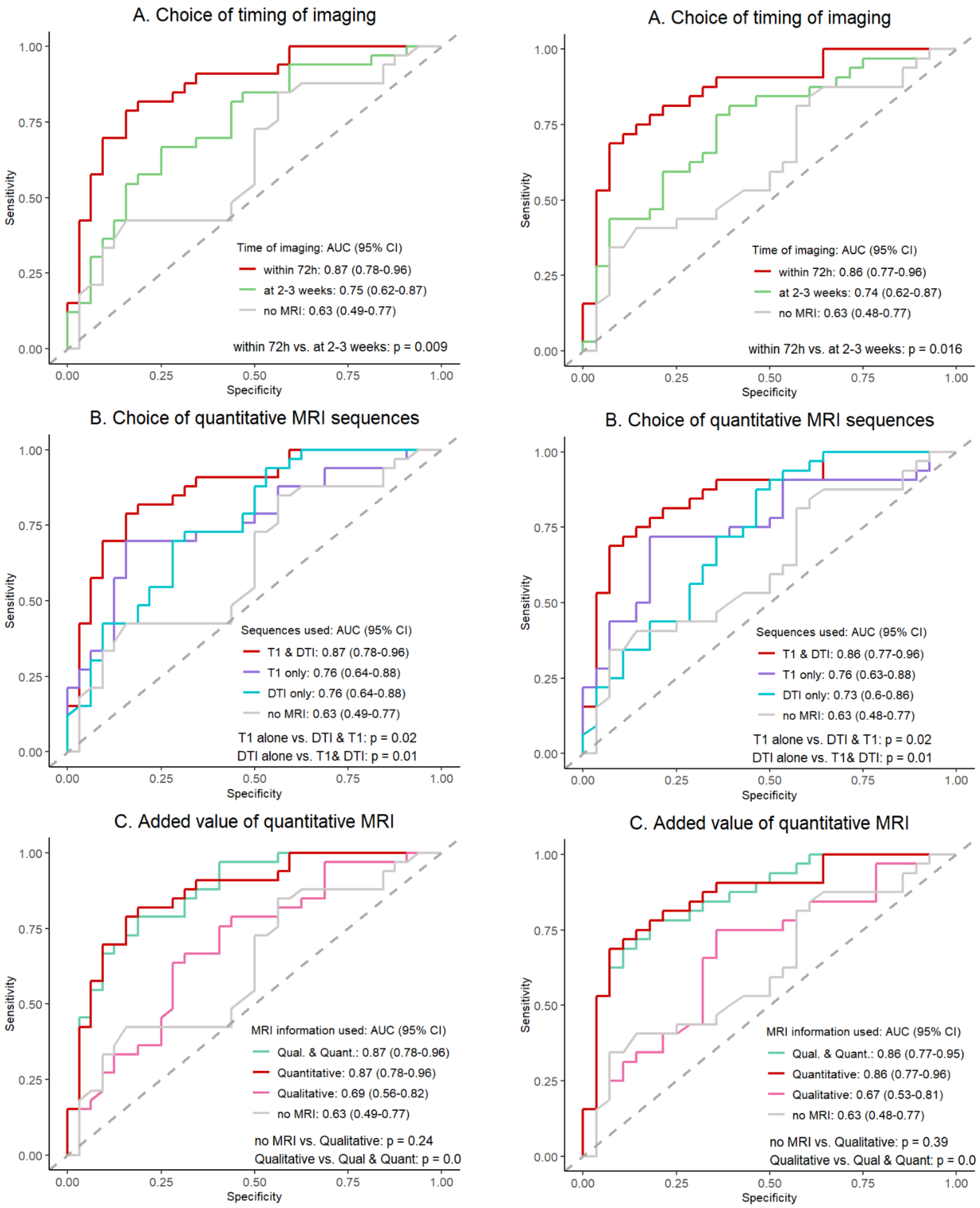

Left-hand panel: original analysis of all mild TBI patients presented as Figure 2 in the main manuscript. Right-hand panel: sensitivity analysis after exclusion of patients with visible mass lesions on their initial computed tomography scan (CT) i.e. a Marshall score of 5 or 6.

©2021 Richter S et al. *JAMA Network Open*.
